# Crystallographic Analysis of Human Serum Albumin Complexed with 4*Z*,15*E*-Bilirubin-IXα

**DOI:** 10.1016/j.jmb.2008.06.016

**Published:** 2008-08-29

**Authors:** Patricia A. Zunszain, Jamie Ghuman, Antony F. McDonagh, Stephen Curry

**Affiliations:** 1 Biophysics Section, Blackett Laboratory, Imperial College, South Kensington Campus, Exhibition Road, London SW7 2AZ, UK; 2 Division of Gastroenterology and the Liver Center, Room S-357, Box 0538, University of California, 513 Parnassus Ave, San Francisco, CA 94143-0538, USA

**Keywords:** HSA, human serum albumin, human serum albumin, bilirubin, crystal structure, fusidic acid, ligand binding

## Abstract

Bilirubin, an insoluble yellow-orange pigment derived from heme catabolism, accumulates to toxic levels in individuals with impaired or immature liver function. The resulting jaundice may be managed with phototherapy to isomerize the biosynthetic 4*Z*,15*Z*-bilirubin-IXα to more soluble and excretable isomers, such as 4*Z*,15*E*-bilirubin. Bilirubin and its configurational isomers are transported to the liver by human serum albumin (HSA) but their precise binding location(s) on the protein have yet to be determined. To investigate the molecular details of their interaction, we co-crystallised bilirubin with HSA. Strikingly, the crystal structure—determined to 2.42 Å resolution—revealed the 4*Z*,15*E*-bilirubin-IXα isomer bound to an L-shaped pocket in sub-domain IB. We also determined the co-crystal structure of HSA complexed with fusidic acid, an antibiotic that competitively displaces bilirubin from the protein, and showed that it binds to the same pocket. These results provide the first crystal structure of a natural bilirubin pigment bound to serum albumin, challenge some of the present conceptions about HSA–bilirubin interactions, and provide a sound structural framework for finally resolving the long-standing question of where 4*Z*,15*Z*-bilirubin-IXα binds to the protein.

## Introduction

**Fig. 1:**
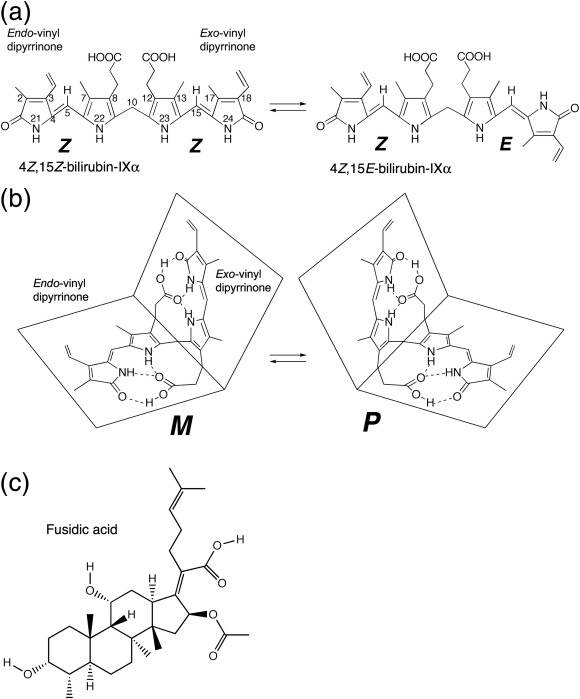
Structure and stereochemistry of bilirubin and fusidic acid. (a) Structures of 4*Z*,15*Z*-bilirubin-IXα  and the photoisomer, 4*Z*,15*E*-bilirubin-IXα, that is observed bound to HSA in this study. Other possible configurational photoisomers (4*E*,15*Z*-bilirubin and 4*E*,15*E*-bilirubin) are not shown. (b) Structures of the *M* and *P* enantiomers of 4*Z*,15*Z*-bilirubin-IXα in the “ridge-tile” conformation. (c) Structure of fusidic acid.

Bilirubin†, an orange-yellow pigment that results from the breakdown of heme, is produced at a rate of about 300 mg per day in human adults. The biosynthetic form of the bilirubin molecule (4*Z*,15*Z*-bilirubin IXα; Fig. 1a), which is lipophilic and insoluble in water at neutral pH, is transported in blood plasma by serum albumin to the liver where it is converted to a water-soluble glucuronide that is excreted into bile and ultimately eliminated via the intestine.

The lipophilicity and poor aqueous solubility of bilirubin was once something of a paradox, since the molecule is a dicarboxylic acid containing several polar functional groups. The paradox was resolved when the crystal structure of the compound was determined^1^ and revealed that the molecule adopts a “ridge-tile” conformation in which the interplanar angle between the dipyrrinone groups is about 100°, allowing each of the two propionic acid groups to make three hydrogen bonds with the opposite dipyrrinone ring system and preventing them from interacting with water (Fig. 1b). Later work showed that the ridge-tile conformation occurs in solution, although the bilirubin molecule can flip between two enantiomeric conformers (*P* or *M*), both of which maintain the internal hydrogen bond network^2^ (Fig. 1b). Further evidence of the flexibility of the molecule came from the crystal structure of the di-isopropylammonium salt of bilirubin.^3,4^ In the salt, the ridge-tile conformation is largely retained, but some of the intramolecular hydrogen bonds are replaced by interactions with isopropylammonium counterions. A similar exchange of intramolecular for intermolecular interactions is believed to occur on binding of bilirubin to albumin,^4^ since thermodynamic evidence shows that electrostatic interactions are important for protein binding.^5^ Evidence from circular dichroism studies shows that binding of bilirubin to human serum albumin (HSA) is enantioselective, since the *P* enantiomer is bound preferentially;^6,7^ however, precise details of the conformation adopted by bilirubin when bound to the protein are lacking.

This issue is complicated by the photoisomerization of bilirubin, a process that is relevant to the clinical management of metabolic disorders involving the pigment. Bilirubin is normally present in plasma at low concentration (5–17 μM) but, when conjugation of bilirubin is deficient, the unconjugated pigment accumulates in the circulation and extravascular tissue, where it may become visually detectable (jaundice). Jaundice is common in newborn infants because of a transient developmental deficiency of conjugation combined with increased hemoglobin breakdown.^8^ In severe cases, the bilirubin-binding capacity of plasma albumin may be exceeded, allowing sufficient bilirubin to cross the blood–brain barrier and cause permanent neurological damage (kernicterus). Phototherapy with blue or white light is commonly used in the clinical management of neonatal jaundice. The treatment enhances the elimination of bilirubin by photochemically converting it to more polar photoisomers and low molecular weight fragments that do not require conjugation with glucuronic acid for excretion. Two types of photoisomers are formed; structural and configurational. In the latter, rotation around the *Z* configuration C = C double bond within one or both of the dipyrrinone moieties results in a configurational change that partially or completely disrupts the internal hydrogen bonding, thereby enhancing the solubility and rate of secretion into the bile (Fig. 1a) The reaction is reversible, and since the four configurational isomers have similar absorbance spectra, irradiation of bilirubin with broad-spectrum light *in vitro* eventually leads to a photostationary mixture of all four in which the 4*Z*,15*Z* isomer invariably predominates.^9^

Configurational photoisomerization of bilirubin bound to HSA is highly regioselective for the C15 double bond and generates mainly the 4*Z*,15*E* isomer.^10^ Although *E* photoisomers of bilirubin are unstable in protic solvents, their thermal reversion to the *Z*,*Z* isomer in the dark is very slow when they are bound to HSA. The 4*Z*,15*E*-isomer is also the predominant photoisomer present in the blood of neonates undergoing phototherapy and in the blood of normal adults after exposure to sunlight.^8,11^ Although 4*Z*,15*E*-bilirubin clearly interacts with HSA, little is known about its locus or affinity of binding.

HSA, the primary transport vehicle for bilirubin species in blood, is a highly soluble protein present in plasma at a concentration of around 0.6 mM in healthy adults. The monomeric protein is composed of three structurally homologous helical domains (I, II and III), each of which is divided into two sub-domains (A and B).^12^ HSA preferentially binds lipophilic anions and serves as a transporter for a variety of small-molecule ligands. Its primary cargo appears to be non-esterified fatty acids but the protein is also known to have a high affinity for other endogenous anions, such as bilirubin, heme and thyroxine.^13^ Moreover, HSA has a well-characterized affinity for lipophilic drug molecules that have anionic or electronegative features.^13^

The 4Z,15Z-isomer of bilirubin binds with high affinity (*K*_D_ ∼ 16 nM) to a single site on HSA.^14^ There are also one or two secondary sites with affinities that are at least tenfold lower.^15^ These are thought to be unimportant *in vivo* but can complicate the interpretation of *in vitro* binding experiments.^16^ Despite numerous studies, the location of the primary bilirubin-binding site on albumin is not known. There is evidence that it is not in drug site 2, within sub-domain IIIA,^17^ but evidence in favor of either the heme-binding site in sub-domain IB^18,19^ or the drug-binding site 1 in subdomain IIA^20–22^ is equivocal and contradictory.

To investigate the molecular details of the binding of this important metabolite to albumin we complexed bilirubin with HSA and determined the co-crystal structure. We found that it binds to a primary site in sub-domain IB of the protein but curiously, the electron density indicates that the bound pigment is predominantly the 4*Z*,15*E* isomer in a distorted ridge-tile conformation, probably formed via adventitious exposure of the 4*Z*,15*Z* complex to light during the course of our experiments. We also determined the co-crystal structure of HSA complexed with the antibiotic fusidic acid, which has been reported to compete with 4*Z*,15*Z*-bilirubin for binding to albumin *in vitro*,^23^ and observed that it occupies the same binding site in sub-domain IB as 4*Z*,15*E*-bilirubin. The results identify a new marker for binding to domain IB and provide the first evidence for the binding site of the clinically important 4*Z*,15*E* photoisomer of bilirubin to HSA. They also raise interesting questions about the location on the protein of the primary binding site of the biosynthetic 4*Z*,15*Z* isomer and suggest new ways in which they might be resolved.

## Results

### Structure determination of the HSA-bilirubin complex

**Fig. 2:**
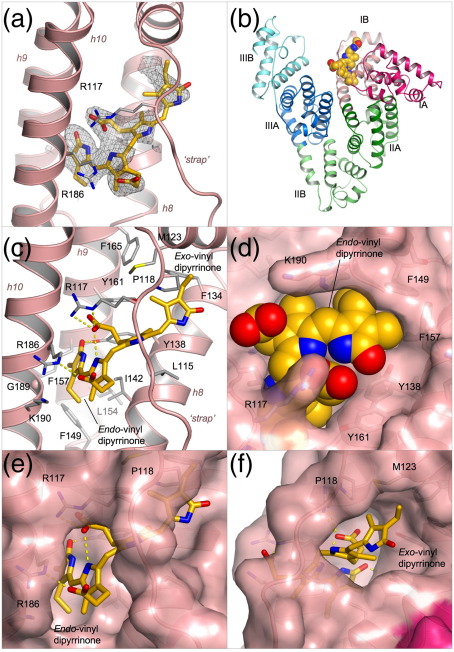
Crystal structure of HSA complexed with 4*Z*,15*E*-bilirubin-IXα. (a) Simulated annealing *F*_o_–*F*_c_ omit map contoured at 1.75σ showing the pigment bound to sub-domain IB of HSA. 4*Z*,15*E*-bilirubin is shown in a stick representation with atoms coloured by atom-type: C, yellow-orange; O, red; N, blue. Sub-domain IB is shown in a ribbon representation (light red). (b) Overall structure of HSA complexed with 4*Z*,15*E*-bilirubin. The pigment is depicted with space-filling spheres; the protein secondary structure is shown colored by sub-domain. (c) Detailed view of the interactions of 4*Z*,15*E*-bilirubin with the binding pocket in sub-domain IB. Hydrogen bonds are indicated by broken yellow lines. Selected protein sidechains are shown as sticks (with carbon atoms colored grey). (d) View of the fit of 4*Z*,15*E*-bilirubin (shown as a space-filling CPK model) to the pocket in sub-domain IB. (e) View of the fit of 4*Z*,15*E*-bilirubin to the contours of the binding pocket in sub-domain IB (shown as a pink, semi-transparent surface). (f) Same as in d but rotated ∼90° about a vertical axis.

Bilirubin was incubated in the dark with HSA at a 1.1:1 molar ratio of ligand to protein and co-crystallised in a P1 unit cell that contains two molecules in the asymmetric unit and is the same as that obtained with other HSA–ligand complexes.^24,25^ A complete X-ray diffraction dataset was collected to 2.42 Å and phased by molecular replacement (Materials and Methods). Following initial rigid body refinement, calculation of a difference electron density map gave a clear indication of a single tetrapyrrole molecule bound to sub-domain IB of the protein (Fig. 2a and b). There was also fragmentary positive difference electron density in drug sites 1 and 2 (in sub-domains IIA and IIIA, respectively), but in neither site was the density sufficient to guide the building of even one dipyrrinone moiety of bilirubin. In subsequent experiments, HSA-bilirubin was co-crystallised from a 3:1 molar ratio of bilirubin to HSA. The structure of this complex was solved to 2.9 Å resolution (data not shown); the electron density map clearly confirmed the presence of pigment in sub-domain IB but again contained only fragmentary electron density in sub-domains IIA and IIIA that could not be interpreted in molecular terms.

In contrast, the density in sub-domain IB gave a good indication of the disposition of the dipyrrinone chromophores and propionate side-chains of the bound ligand (Fig. 2a). It was immediately obvious that the ridge-tile conformation of 4*Z*,15*Z*-bilirubin IXα that occurs in solution (Fig. 1b) could not fit the density.^2,26^ Even if we allowed rotation around the C9-C10 and C10-C11 bonds to disrupt intramolecular interactions and produce a more extended conformation, it was not possible to generate a reasonable fit; while this manipulation allowed a match to three of the heterocyclic rings, it could not account for the density corresponding to the remaining ring. Instead, the electron density suggested that one of the dipyrrinone groups had adopted an *E* configuration; the best fit was obtained by building the 4*Z*,15*E* isomer of bilirubin in an orientation that placed the *endo*-vinyl dipyrrinone moiety (with the *Z* configuration) in a predominantly hydrophobic cleft generated by the packing of helices h8–h10 into a three-helix bundle (Fig. 2c), while the *exo*-vinyl dipyrrinone moiety (with the *E* configuration and twisted periplanar or synclinal conformation) was accommodated in a more open section of the pocket, packed against helices h7-h8 and pinned underneath the polypeptide linker (residues 110–119) that connects IB with the preceding sub-domain (Fig. 2c). This model provides the best fit of the methyl and vinyl groups on the planar *endo*-vinyl dipyrrinone group to the observed density; it also allows both rings of the *exo*-vinyl dipyrrinone moiety to be placed in density, though the precise orientation of the outer lactam ring is not clearly defined (Fig. 2a). It is noteworthy that the *M* helicity of the bound 4*Z*,15*E* isomer is consistent with CD measurements on the pigment in the presence of HSA. (A. F. M., unpublished results).

Since the density for the bound pigment at the current map resolution is not completely contiguous, we cannot rule out the possibility that the 4*E*,15*Z* isomer may be present also in some fraction of the HSA molecules in the crystal. Nevertheless, the interpretation that the 4*Z*,15*E* isomer is the predominant isomer bound within the crystal structure is consistent with the observation that binding to HSA favors photoisomerization of bilirubin to the 4*Z*,15*E* form.^10^

Although the 4*Z*,15*E* isomer provides the best fit to the observed density and a plausible model for 4*Z*,15*Z*-bilirubin could not be made, the crystallographic data do not exclude the possibility that some fraction of the bound pigment is present in this form. Modeling studies indicate that 4*Z*,15*Z*-bilirubin may be able to fit within the binding site in sub-domain IB in a partially extended conformation in which the interplanar angle between the dipyrrinone groups is increased from 100°, allowing the molecule to adopt an overall shape that is broadly similar to that built for the 4*Z*,15*E* isomer (though some adjustments of the pocket side-chains would be needed to avoid steric clashes). However, this modeled conformation has *M*-type helicity, which is opposite to the *P*-type helicity observed in CD measurements of 4*Z*,15*Z*-bilirubin bound to HSA. The source of this inconsistency is not clear; although CD measurements are very sensitive to conformational changes in bilirubin, it is still difficult to derive a precise model of protein-bound bilirubin conformations using CD data.^7,27^

The origin of the unexpected 4*Z*,15*E* photoisomer is most likely intermittent exposure of the HSA–bilirubin samples to ambient light during crystallization, crystal manipulation and X-ray data collection. The original bilirubin used for the experiments contained <2% 4*Z*,15*E*-bilirubin as determined by HPLC analysis (data not shown). Control studies showed that 4*Z*,15*Z*-bilirubin does not isomerize to 4*Z*,15*E*-bilirubin during several days incubation with excess HSA in the dark at 37 °C. Although precautions were taken to exclude light, samples were exposed intermittently to light during solution preparation and particularly during microscopic examination. HPLC analyses of crystallization samples (crystals and mother liquor) confirmed the presence of the 4*Z*,15*E* photoisomer at concentrations of up to ∼20% that of the 4*Z*,15*Z* isomer (data not shown).

### Structure of HSA complexed with 4*Z*,15*E*-bilirubin-IXα

The crystal structure of HSA complexed with 4*Z*,15*E*-bilirubin was fully refined to yield a model with an *R*_free_ of 27.4% with good stereochemistry (Table 1). The pigment is bound within a central channel formed by all four helices of sub-domain IB and is evidently ”strapped” into place by the extended polypeptide that connects this sub-domain with sub-domain IA (residues 110–119) (Fig. 2) and by the formation of salt-bridges with a pair of arginine residues, R117 and R186, which lie at the entrance to the binding cleft (Fig. 2c). Binding is accompanied by only minor conformational changes in some of the side-chains lining the binding site, whereas the pigment is significantly distorted from the ridge-tile conformation (though it broadly maintains an L-shaped conformation).

The *endo*-vinyl dipyrrinone group, containing the 4*Z* double bond, maintains a *syn-planar* conformation and is accommodated in the deepest part of the binding cleft, largely shielded from solvent. It makes close contacts with the apolar side-chains of residues from helices h8–h10 (I142, F149, L154, F157, G189 and the aliphatic portion of K190) (Fig. 2c–e). The carbonyl oxygen of the lactam ring is hydrogen-bonded to the side-chain of Y138 (2.6 Å), though neither of the two nitrogen atoms of the *endo*-vinyl dipyrrinone group makes specific interactions with the protein. Only one of the three intramolecular hydrogen bonds made by this dipyrrinone group in solution is retained in the bound conformation—from the nitrogen on the pyrrole ring to the propionate carboxylate group of the *exo*-vinyl dipyrrinone group. This carboxylate group also makes a salt-bridge interaction with the side-chain of R117 (2.7 Å). The carboxylate group attached to the *endo*-vinyl dipyrrinone group is in fact directed away from the *exo-*vinyl dipyrrinone and makes no intramolecular interaction when bound to HSA; instead, although partially shielded from solvent, it is oriented towards the entrance to the pocket and makes a salt-bridge to the side-chain of R186 (2.7 Å) (Fig. 2c).

A planar conformation for the *exo*-vinyl dipyrrinone group that contains the 15*E* double bond is unfavorable because of steric interactions of the methyl substituents. As predicted by force-field calculations,^28^ it occurs in a twisted *syn*-periplanar conformation in a more exposed part of the cleft formed by the polypeptide ”strap” and helices h7 and h8 (Fig. 2c and f). The inner pyrrole is bound directly underneath the polypeptide strap, pinned by apolar contacts with the side-chains of L115 and R117, while the outer lactam ring is more exposed to solvent. The *E* configuration allows the methyl and vinyl substituents on the lactam ring to pack against the hydrophobic flank of this part of the pocket (composed largely of the side-chains of P118, M123, F134 and F165) but its amide and carbonyl groups are exposed to solvent and appear to make no contact with the protein. The NH of the inner pyrrole is 3.1 Å from the carbonyl oxygen of L115 but the geometry for hydrogen bond formation is poor. Thus the *exo*-vinyl dipyrrinone moiety of 4*Z*,15*E*-bilirubin makes no specific hydrogen bond interactions with the protein.

### Structure of the HSA–fusidic acid complex

**Fig. 3:**
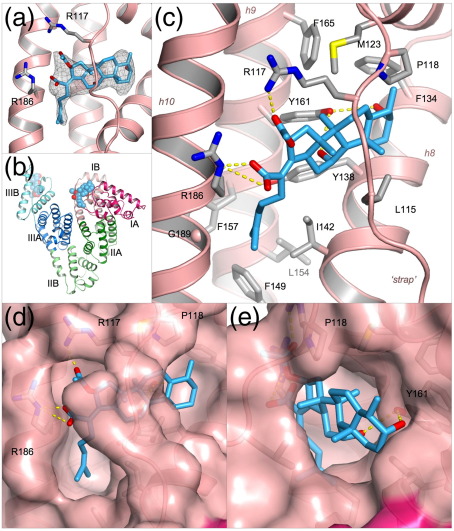
Crystal structure of HSA complexed with fusidic acid. (a) Simulated annealing *F*_o_ – *F*_c_ omit map contoured at 1.75σ showing the drug bound to sub-domain IB of HSA. Fusidic acid is shown in a stick representation with atoms colored by atom-type: C, light blue; O, red; N blue. (b) Overall structure of HSA complexed with fusidic acid. The drug, depicted with space-filling spheres, binds to sub-domains IB and IIIB. (c) Detailed view of the interactions of fusidic acid with the binding pocket in sub-domain IB. Hydrogen bonds are indicated by broken yellow lines. Selected protein sidechains are shown as sticks (with carbon atoms colored grey). (d) View of the fit of fusidic acid to the binding pocket in sub-domain IB (pink, semi-transparent surface). (e) Same as in d but rotated ∼90° about a vertical axis.

Given the uncertainty in the literature regarding the location of bilirubin binding on HSA, we sought additional experimental support for the location of the primary site. There is *in vitro* evidence that fusidic acid competes with 4*Z*,15*Z*-bilirubin for binding to HSA,^23^ suggesting that both may bind to the same pocket on the protein. The co-crystal structure of HSA-fusidic acid was determined to 3.05 Å (Materials and Methods) and refined to yield a model with an *R*_free_ of 27.0% (Table 1). The crystal structure reveals the presence of two fusidic acid molecules associated with HSA (Fig. 3a and b). One of these is bound to the outer surface of sub-domain IIIB but also makes contact with a symmetry-related HSA molecule in the crystal, suggesting that this site is therefore likely to be an artefact of co-crystallisation. The other fusidic acid molecule is bound entirely within sub-domain IB (Fig. 3b).

Strikingly, the fusidic acid adopts an L-shaped conformation and occupies the binding pocket in sub-domain IB in a manner very similar to that of 4*Z*,15*E*-bilirubin (Fig. 3c). The steroid moiety is found in the more open section of the binding pocket, lying directly underneath the polypeptide strap and in essentially the same position as the *exo*-vinyl dipyrrinone group of 4*Z*,15*E*-bilirubin (Fig. 3d and e). The side-chain hydroxyl of Y161 makes equidistant hydrogen bonds (3.3 Å) with the two hydroxyl groups that project from the steroid rings of fusidic acid. Towards the other end of the drug molecule, the hydrocarbon tail of its methyl-heptenoic acid group projects into the deeper part of the binding pocket that was observed to be occupied by the *endo*-vinyl dipyrrinone group of 4*Z*,15*E*-bilirubin (Fig. 3c and d), but does not penetrate quite as far into this cavity; thus derivatives of fusidic acid with larger apolar substituents in this region might be expected to bind with higher affinity. The acetyloxy and carboxylate groups of fusidic acid are positioned at the entrance to the pocket where they make hydrogen bond interactions with R117 and R186 respectively (Fig. 3c), the same arginine residues that interact with the carboxylate groups of 4*Z*,15*E*-bilirubin. Notably, both of the Arg side-chains are significantly re-oriented in order to establish these specific interactions with the drug; an upward rotation of the side-chain of R117 by about 40° is required to provide room to accommodate the increased bulk of the steroid moiety of fusidic acid. Elsewhere, there are only minor differences in protein side-chain conformations between the HSA complexes with 4*Z*,15*E*-bilirubin and fusidic acid (primarily involving L182, M123) and a small (0.8 Å) displacement of the polypeptide strap.

## Discussion

Our crystallographic analysis of the complex formed by incubating bilirubin with HSA unexpectedly yielded the structure of the clinically important 4*Z*,15*E*-bilirubin–HSA complex but nevertheless provided intriguing new insights into the molecular details of how this toxic bile pigment interacts with its primary transporter.

With hindsight, it is clear that precautions to exclude light were insufficiently rigorous and that the 4*Z*,15*E*-isomer found in the crystals used for structure determination was produced photochemically with each exposure of the crystallization mixture to ambient light, particularly during repeated microscopic examination of the sitting drops. Even though the binding of bilirubin to HSA is known to favor photoisomerization to the 4*Z*,15*E* form, the crystallographic result is still something of a surprise, because experiments in solution suggest that this form does not usually exceed 40% of the bilirubin present due to rapid photo-equilibration between the 4*Z*,15*Z* and 4*Z*,15*E* forms.^10^ One possible explanation for our findings is that the 4*Z*,15*E*-bilirubin isomer becomes locked in the binding site in sub-domain IB due to close contacts between proteins in the crystal. Earlier, we observed that ligands that bind reversibly to HSA in solution can become trapped in crystalline samples; for example, thyroxine remained bound to its high-affinity site in sub-domain IIA,^29^ even after back-soaking the HSA–thyroxine crystals against thyroxine-free solutions for periods in excess of 48 h (data not shown). However, further work is required to fully explain the formation and isolation of crystalline HSA with the 4*Z*,15*E* form of bilirubin within site IB. Interestingly, the crystal structure of the *Z,E* isomer of a symmetrically substituted synthetic bilirubin has been reported recently.^30^ This isomer also appears to have been formed by incidental exposure of solutions to light during attempts to crystallize the corresponding *Z,Z* isomer.

If the 4*Z*,15*E* bilirubin observed in the crystal in sub-domain IB was generated *in situ* from 4*Z*,15*Z* bilirubin bound at the same site, the structure would provide an explanation for the observation that photo-isomerization of bilirubin bound to HSA is highly regioselective for the exo-vinyl dipyrrinone.^10^ The crystal structure shows that the *endo*-vinyl dipyrrinone moiety, which retains its planar Z conformation during this photoisomerization, fits snugly into the deep part of the binding cavity formed by helices h8–h10. In particular, the contours of the pocket appear well matched to the arrangement of the methyl and vinyl groups that project from adjacent carbon atoms of the outer lactam ring (Fig. 2d). In contrast, the *exo*-vinyl dipyrrinone moiety is held less tightly in a more open section of the binding site (formed by the polypeptide strap and helices h7-h8) that permits accommodation of the twisted E conformation observed for the 4*Z*,15*E* isomer. The structure suggests that the alternate 4*E*,15*Z*-isomer may bind less well, assuming that the bound conformation is flipped to place the planar *exo*-vinyl dipyrrinone group (*Z* conformation) in the more enclosed portion of the binding cavity. This is because the arrangement of the methyl and vinyl groups on the outer lactam ring of the *endo*-vinyl dipyrrinone group is the reverse of that on the *exo*-vinyl dipyrrinone group and does not therefore allow a precise fit inside the cavity; indeed, we would predict a steric clash between the vinyl group and the side-chain of F157. The structure thus suggests that photoisomerization to the 4*Z*,15*E* form would be favored by binding of 4*Z*,15*Z*-bilirubin to sub-domain 1B because the open section of the pocket has sufficient room and flexibility to permit rotation of the *exo*-vinyl lactam ring on photoexcitation.

### Is sub-domain IB the primary binding site for 4*Z*,15*Z*-bilirubin IXα on HSA?

This is a key question, since the 4*Z*,15*Z*-isomer of bilirubin is the predominant form *in vivo*.^31^ From our data, it is impossible to deduce whether the 4*Z*,15*E*-bilirubin observed in the crystal was formed from 4*Z*,15*Z*-bilirubin initially bound to the same site or whether it was formed by photoisomerization of 4*Z*,15*Z*-bilirubin bound to another site followed by migration of the 4*Z*,15*E* isomer to subdomain IB.

**Fig. 4:**
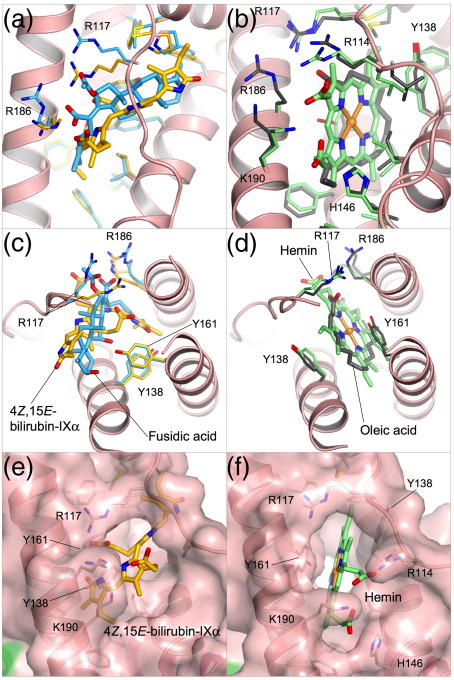
The flexible binding capacity of sub-domain IB in HSA. (a, c) Comparative binding of 4*Z*,15*E*-bilirubin and fusidic acid. Ligands are shown in a stick representation with yellow-orange (4*Z*,15*E*-bilirubin) or light-blue (fusidic acid) carbon atoms. (b, d) Comparative binding of oleic acid^55^ and hemin.^45^ Ligands are shown in a stick representation with dark grey (oleic acid) or light-green (hemin) carbon atoms. (c and d) Top view relative to the orientation shown in (a) and (b). Variation in the geometry of the binding pocket in sub-domain IB is evident from the surface representation of the protein shown for the complex with 4*Z*,15*E*-bilirubin (e) and the complex with hemin (f). Binding of hemin, but not 4*Z*,15*E*-bilirubin, induces large rotations of Y138 and Y161 which open up a D-shaped cavity that extends to the back of the sub-domain.

Although the new crystallographic data presented here supply no direct evidence for the hypothesis that 4*Z*,15*Z*-bilirubin IXα binds primarily to sub-domain IB of HSA, they offer some indirect support that is worth considering. The crystal structure clearly shows that sub-domain IB contains a pre-formed L-shaped binding cavity that is the appropriate size to accommodate the 4*Z*,15*E* isomer of bilirubin (Figs. 1a, and 2e and f). Modeling studies suggest that binding of the 4*Z*,15*Z*-isomer could be achieved in the same pocket with only relatively minor adjustments of the pigment (to open up the ridge-tile conformation that is observed in solution^2^), and of some of the side-chains and the polypeptide strap in sub-domain IB. Comparison of the HSA-bilirubin and HSA-fusidic acid complexes shows that the flexibility of side-chains lining the pocket confers considerable adaptability, allowing it to form specific interactions with different ligands (Fig 4a and c; see below). Moreover, the observation that fusidic acid binds specifically to sub-domain IB provides a plausible explanation for the observation that this drug competes with bilirubin—presumed to be in its 4*Z*,15*Z* form—for binding to the protein.^23^

Nevertheless, the location of the primary binding site for bilirubin on HSA remains to be established. Although the most recent reviews that have considered the question have tended to highlight sub-domain IIA (drug site 1) as the primary binding site for bilirubin,^13,32^ the evidence accumulated over many years is actually rather equivocal on this point.

One of the earliest attempts to locate binding sites for bilirubin on albumin compared the binding affinity of a range of proteolytic fragments of BSA.^22^ Such experiments can be difficult to interpret even when the structure of the intact protein is known, because the structural impact of proteolysis is not predictable. The authors of the study identified residues 186–238, which comprise most of sub-domain IIA, as a region common to fragments that retained high-affinity binding. However, this interpretation may under-estimate the significance of the large variations in affinity between fragments containing this region. For example, although fragment 186–306 (sub-domain IIA) had a *K*_A_ of 5.4 × 10^6^ M^−1^, this is about tenfold lower than the bilirubin-binding affinity of the fragment 1–306 (sub-domains IA-IB-IIA). This could suggest that locations outside sub-domain IIA may contribute significantly to high-affinity binding.

Other approaches probed the ability of albumin-binding drugs to displace bilirubin from the protein as a means to localize its binding site. This method has the advantage of using intact protein, although the presence of primary and secondary binding sites for the competing ligands can complicate the interpretation of the results. One study of this type showed that diazepam, a drug site 2 compound that binds specifically and uniquely to sub-domain IIIA,^24^ did not displace bilirubin from its primary site.^17^ This result, which was the first demonstration of co-binding of two ligands to a single polypeptide, suggests that bilirubin does not bind primarily to sub-domain IIIA. Against this, it has been found that ibuprofen—another drug that binds primarily to drug site 2—can displace bilirubin from HSA.^33^ A precise interpretation of this latter result is difficult, since recent data show that ibuprofen also binds to two secondary sites in sub-domain II, one of which is co-incident with drug site 1.^24^

Investigations using compounds that bind specifically to drug site 1 in sub-domain IIA have produced results that are non-trivial to interpret at the molecular level. Thus warfarin, phenylbutazone, azapropazone, indomethacin, iophenoxic acid and thyroxine were all found to displace bilirubin from the protein,^20,23,34–37^ findings that at first sight are consistent with sub-domain IIA being the primary locus of bilirubin binding. However, in some cases displacement of bilirubin was observed only at high concentrations of competitor,^20,34–36^ which raises the possibility that displacement of bilirubin may be due to drug binding at secondary sites outside sub-domain IIA. In fact, Fehske and colleagues concluded that the site 1 drugs warfarin, azapropazone and phenylbutazone did not compete with the primary bilirubin site. For several of the competitor compounds used in these studies (warfarin, azapropazone, indomethacin and iophenoxic acid), crystallographic analysis has revealed secondary binding sites in sub-domain IB^24,38^ (A. Ryan and S. C., unpublished results) which is consistent with the notion that some of the displacement activity may be attributed to the binding of bilirubin to this site.

More recent investigations used site-directed mutagenesis to test the hypothesis that sub-domain IIA is the primary binding site for bilirubin. Eight different amino acid substitutions were introduced into sub-domain IIA but in no case was a significant reduction in the binding affinity observed,^21^ suggesting that the pigment binds elsewhere on the protein. This result is in contrast to a parallel investigation that found some of the same mutations caused a significant decrease in the binding affinity of warfarin,^39^ a drug that is known to bind within sub-domain IIA. Nevertheless, several of the mutations in IIA had a very marked impact on the CD spectra obtained from HSA–bilirubin complexes,^21^ consistent with the notion that bilirubin binds primarily within that sub-domain. As yet, it has not been possible to reconcile these apparently divergent observations, though it is intriguing to note reports of possible allosteric interactions between the heme binding site in sub-domain IB and drug site 1 in sub-domain IIA.^40^

Experiments that have addressed the question of competition between bilirubin and hemin directly have generally found that these molecules can bind to albumin independently,^18,41,42^, although some competition was observed at high ligand to HSA molar ratios.^19^ Since the hemin-binding site on albumin has been located unequivocally in sub-domain IB,^43–45^ these data therefore suggest that the primary bilirubin-binding site on the protein lies outside sub-domain IB. Curiously, one of these studies found that hemin competed with MADDS (monoacetyldiaminodiphenyl sulphone),^18^ a compound that has been shown to be an effective marker for the high-affinity site for bilirubin (and is displaced by fusidic acid).^23^ The interaction between hemin and bilirubin on HSA clearly warrants further investigation.

Overall, it has not been possible to arrive at a definitive assignment of the location of the high-affinity binding site for 4*Z*,15*Z*-bilirubin on HSA. Our results at least raise the interesting possibility that the binding site is located within sub-domain IB. This would readily explain the displacement of bilirubin by fusidic acid, which we have also shown to bind to sub-domain IB. It is consistent also with some of the existing literature on bilirubin binding, but—as discussed above—there are many observations that conflict with this hypothesis. Although we observed only weak difference density in sub-domain IIA that was insufficient to allow modeling of bilirubin at this site, other crystallographic data show how molecules that are similar in dimensions to bilirubin, such as iodipamide, might be accommodated in this sub-domain.^24^

The structural information presented in this report will allow the design of new experimental approaches to determine the site of bilirubin binding. For example, it would be interesting to examine the effect of mutating residues in sub-domain IB that are observed to contact 4*Z*,15*E*-bilirubin-IXα (e.g. L115, R117, Y138, F157, and G189) to determine if they have an effect on the binding of the 4Z,15Z isomer. Moreover, the accumulating database of crystal structures of HSA–drug complexes^24,38,46^ has engendered a much more detailed understanding of the binding locations of a wide range of small molecule compounds that might now be used for more finely controlled competition binding experiments. Further efforts to obtain crystals of HSA complexed with 4*Z*,15*Z* bilirubin should also be informative, although our experience indicates that preparation and crystallization of the complex is likely to require stringent working under orange or red safelights to prevent the formation of the 4Z,15E isomer. This approach was used successfully in the determination of the structure of rhodopsin while maintaining its chromophore, retinol, in the ground state.^47^

### Sub-domain IB on HSA provides a flexible and versatile binding pocket

The structural data on HSA complexes with 4*Z*,15*E*-bilirubin and fusidic acid provide fresh insight into the versatile binding capacity of this remarkable transport protein. Both ligands bind into an essentially pre-formed L-shaped cavity within sub-domain IB, their interactions with the protein largely involving side-chain adjustments. The mode of binding of these two ligands to HSA contrasts markedly with the binding of other compounds such as fatty acids or hemin, both of which induce a significant conformational rearrangement of sub-domain IB (Fig. 4). In particular, binding of fatty acids or hemin causes the side-chains of Y138 and Y161 to un-stack, each tyrosine rotating ∼90° to opposite sides of the binding cavity and thereby redefining the pocket geometry (Fig. 4a and b).^45,48,49^ Y138 moves to form the outside flank of the pocket, largely closing off the entrance to the more open section of the cavity that is bound by the *exo*-vinyl dipyrrinone moiety of the rubin (or the steroid rings of fusidic acid); Y161 lies against the other wall of the cavity. In their new positions the aromatic rings of the two tyrosines are placed flat in contact with the bound ligand, like a pair of hands holding it in place (Fig. 4c–f). The newly formed cavity is D-shaped, and can fully accommodate the planar porphyrin ring of hemin. The methylene tails of fatty acids bound to this pocket curve around the back wall; this leaves sufficient room for co-binding of aromatic drugs or drug-like molecules such as indomethacin and tri-iodobenzoic acid.^24,50^

A further intriguing aspect of the flexibility of the binding pocket in sub-domain IB is the rearrangement of the basic residues at the main entrance to interact with the polar features presence on the different ligands. Thus R117 is engaged in interactions with bound fatty acid, 4*Z*,15*E*-bilirubin and fusidic acid (Fig. 4a-d) but in each case varies its side-chain orientation in order to accommodate the variable positions of the polar carboxyl or acetyloxy groups of the different ligands. R186 is observed to interact only with rubin and fusidic acid (Fig. 4a, and c), again adjusting its position to maintain interactions with the ligand. Hemin, by contrast, interacts with neither of these Arg residues; instead its two propionic acid groups are coordinated by R114, H146 and K190 (Fig. 4b and f).

## Materials and Methods

### Preparation and crystallization of the HSA complexes

Recombinant HSA (Recombumin®) obtained from Delta Biotechnology (Nottingham, UK), was defatted and purified by gel-filtration chromatography to be dimer-free essentially as described,^25,50^ and then concentrated to 100 mg/ml (1.5 mM). Bilirubin (Sigma-Aldrich) containing 6% 4*Z*,15*Z*-bilirubin IIIα, 87% 4*Z*,15*Z*-bilirubin IXα and 5% 4*Z*,15*Z*-bilirubin XIIIα and <2% *Z*,*E* isomers (as determined by HPLC)^51^ was dissolved immediately before use at 10 mM in dimethylsulfoxide (DMSO) in a foil-wrapped microcentrifuge tube. The bilirubin and HSA solutions were mixed to give a bilirubin to HSA molar ratio of 1.1:1 and 3:1, and incubated with rotation in the dark overnight at room temperature. Finally, the complex was washed with repeated cycles of concentration and dilution with 50 mM potassium phosphate buffer (pH 7.0) using a 5 kDa cut-off ultrafiltration device (Vivaspin, Millipore) to reduce the final concentration of DMSO to <0.1% (v/v).

The exposure of HSA–bilirubin solutions and crystals to light was minimized throughout our procedures but not eliminated. Manipulation of solutions (e.g. mixing of HSA and bilirubin or setting up crystallization trials) was performed under ambient natural light but all long-term incubations were performed in foil-wrapped containers stored in a light-proof refrigerator. Periodically, crystallization trials were inspected briefly under a microscope with dim illumination. Crystals were mounted in capillaries under the same conditions and then transported to the synchrotron in foil-wrapped containers. Immediately before data collection, the crystal was mounted on the X-ray camera under ambient light which was then turned off for the 45–90 min required to collect an X-ray diffraction dataset.

Fusidic acid was dissolved at 10 mM in water and mixed with 1.5 mM HSA to obtain a 3:1 molar ratio of fusidic acid to HSA. The resulting complex was concentrated to around 100 mg/ml in a single step by ultrafiltration.

In each case, complexes were analyzed by PAGE to estimate the concentration of protein. Crystals were grown by the sitting-drop, vapor-diffusion method at 4 °C using methods similar to those described elsewhere.^49,50,52^ Crystallization screens were set up at a protein concentration of >90 mg/ml using 20–30% (w/v) PEG 3350 (Sigma-Aldrich) in 50 mM potassium phosphate buffer (pH 7.0) as the precipitant, with sitting drops prepared by mixing 4 μl of the protein solution and 4 μl of the reservoir mixture. Crystals were observed one to three days after streak-seeding, the largest appearing at 24% (w/v) PEG for fusidic acid and 26% (w/v) PEG for bilirubin.

### Data collection, processing, structure determination and refinement

X-ray diffraction data were collected at Daresbury SRS (station 9.6) and EMBL/DESY Hamburg (beamline X11) using crystals mounted in sealed glass capillaries at room temperature at least 24 h before data collection. Mounted crystals were stored at 4 °C and transported in insulated containers chilled with ice-packs. The diffraction data were collected at room temperature, and it was possible to get a full dataset from a single crystal for both complexes. The data were indexed and measured with MOSFLM (Medical Research Council, Laboratory of Molecular Biology, Cambridge, UK) and scaled using the CCP4 suite.^53^ A previously determined structure of HSA (PDB ID code 2bxa^24^) stripped of its ligand was used as a starting model for phasing. Rigid body, positional and *B*-factor refinements were performed using CNS.^54^ Data collection and refinement statistics are summarized in Table 1.

### Protein Data Bank accession numbers

Coordinates and structure factors have been deposited in the Protein Data Bank with accession numbers 2vue (HSA-4*Z*,15*E*-Bilirubin-IXα) and 2vuf (HSA-fusidic acid).

## Figures and Tables

**Table 1:** Data collection and refinement statistics

	HSA-4*Z*,15*E*-bilirubin-IXα	HSA-fusidic acid
A. *Data collection*		
Space-group	*P*1	*P*1
Unit cell parameters		
*a* (Å)	55.09	55.17
*b* (Å)	55.54	55.32
*c* (Å)	119.83	119.83
α (°)	81.22	81.69
β (°)	90.70	90.73
γ (°)	65.42	65.83
Resolution range (Å)	38.07–2.42	35.33–3.05
Independent reflections	45,968	22,450
Multiplicity	1.8 (1.7)	1.9 (1.9)
Completeness (%)	95.0 (85.8)	92.4 (85.5)
*I*/σ_*I*_	9.8 (2.2)	8.9 (1.9)
*R*_merge_ (%)^a^	4.5 (31.0)	7.7 (36.9)

B. *Model refinement*		
Non-hydrogen atoms	8844	8967
*R*_model_ (%)^b^	21.7	23.5
*R*_free_ (%)^c^	27.4	27.0
r.m.s.d. from ideal		
Bond lengths (Å)	0.007	0.008
Bond angles (°)	1.17	1.18
Average *B*-factor (Å^2^)	78.4	58.2
Ramachandran plot	86.5/12.8	85.2/14.5
Most favoured regions (%)		
Allowed regions (%)		
PBD ID	2vue	2vuf

Values in parentheses are for the outermost resolution shell.

a *R*_merge_ = 100 × ∑_*h*_∑_*j*_*|I*_*hj*_*– I*_*h*_*|/*∑_*h*_∑_*j*_*I*_*hj*_, where *I*_*h*_ is the weighted mean intensity of the symmetry-related reflections *I*_*hj*._

b *R*_model_ = 100 × ∑_*hkl*_*|F*_obs_*– F*_calc_*|/*∑_*hkl*_*F*_obs_ where *F*_obs_ and *F*_calc_ are the observed and calculated structure factors, respectively.

c *R*_free_ is *R*_model_ calculated using a randomly selected 5% sample of reflection data omitted from the refinement.

## Footnotes

† Except where specified otherwise, all bilirubin pigments referred to have the IXα substituent constitution. The term “bilirubin” used without qualification refers to the 4*Z*,15*Z*-IXα diastereomer that is the normal end-product of heme catabolism.
